# Facial Nerve Palsy Recovery After Vestibular Schwannoma Surgery: Temporal Patterns and Early Predictors of Functional Outcome

**DOI:** 10.3390/medicina62081614

**Published:** 2026-08-21

**Authors:** Antonio Daloiso, Anna Agostinelli, Giusy Melcarne, Silvia Montino, Stefano Carraro, Stefano Concheri, Giulia Tealdo, Diego Cazzador, Elisabetta Zanoletti

**Affiliations:** 1 Section of Otorhinolaryngology—Head and Neck Surgery, Department of Neuroscience, University of Padova, 35122 Padova, Italy; antoniodaloiso96@gmail.com (A.D.); silvia.montino@unipd.it (S.M.); stefano.carraro_01@studenti.unipd.it (S.C.); stefano.concheri@unipd.it (S.C.); elisabetta.zanoletti@unipd.it (E.Z.); 2 Unit of Otorhinolaryngology—Head and Neck Surgery, Azienda Ospedale-Università Padova, 35128 Padova, Italy; giusy.melcarne@unipd.it (G.M.); giulia.tealdo@aopd.veneto.it (G.T.); diego.cazzador@unipd.it (D.C.)

**Keywords:** facial nerve palsy, facial nerve palsy rehabilitation, neuro-muscular retraining, vestibular schwannoma, lateral skull base

## Abstract

*Background and Objectives*: Facial nerve palsy (FNP) remains a clinically relevant complication after vestibular schwannoma (VS) resection. Recovery trajectories vary according to the severity of neural injury and early postoperative facial function. This study aimed to characterize the temporal pattern of facial nerve recovery during the first postoperative year and to explore factors associated with favorable long-term function. *Materials and Methods*: We retrospectively analyzed 51 adult patients who developed FNP after VS surgery between 2020 and 2023. Facial nerve function was assessed using the House–Brackmann (HB) grading system and the Sunnybrook Facial Grading System (SBFGS) at discharge and at 1, 3, 6, and 12 months postoperatively. Longitudinal changes were analyzed using non-parametric repeated-measures methods with effect-size estimation. Favorable 12-month facial nerve function was defined as HB grade I–II. Given the limited number of unfavorable outcomes, associations with 12-month recovery were explored using parsimonious Firth-penalized logistic regression models. *Results*: At discharge, 45.1% of patients had HB II, 29.4% HB III–IV, and 25.5% HB V–VI. Facial function improved significantly during follow-up. Median SBFGS total score increased from 58.0 at discharge to 99.0 at 12 months (Friedman χ^2^ = 118.78, df = 4, *p* < 0.001; Kendall’s W = 0.58), while HB grade also improved significantly (χ^2^ = 107.48, df = 4, *p* < 0.001; W = 0.53). At 12 months, 37/51 patients (72.5%) achieved HB I–II and none remained in the HB V–VI category. Synkinesis, defined as an SBFGS synkinesis subscore > 0, was observed in 10/51 patients (19.6%) at 6 months and 19/51 (37.3%) at 12 months. HB grade and SBFGS score at discharge were strongly inversely correlated (Spearman ρ = −0.943, *p* < 0.001) and were therefore evaluated in separate models. After adjustment for age and tumor size, each one-grade increase in discharge HB was associated with lower odds of favorable 12-month function (OR 0.30, 95% CI 0.11–0.58; *p* < 0.001), whereas each 10-point increase in discharge SBFGS was associated with higher odds of favorable function (OR 1.90, 95% CI 1.32–3.53; *p* < 0.001). *Conclusions*: Facial nerve function showed substantial longitudinal improvement during the first year after VS surgery. Early postoperative facial function was independently associated with 12-month outcome in exploratory penalized regression analyses. Recovery may continue over several months, supporting prolonged functional surveillance, while the independent contribution of rehabilitation and the effect of nerve grafting require evaluation in larger prospective cohorts.

## 1. Introduction

Despite advances in surgical techniques and intraoperative monitoring, surgical resection of vestibular schwannoma (VS) remains associated with a relevant risk of facial nerve injury [1]. Permanent postoperative facial dysfunction is reported in 10–30% of patients, with rates varying considerably according to tumor size, surgical approach, and center experience [2]. Complete facial nerve transection, requiring immediate surgical reconstruction, occurs in approximately 5–10% of cases, most commonly in the context of large tumors with intimate nerve involvement [3]. Although anatomical preservation of the facial nerve is achieved in the majority of contemporary VS resections, postoperative facial dysfunction remains clinically relevant, particularly after surgery for large or anatomically complex tumors. Complete intraoperative facial nerve disruption is uncommon but represents the most severe form of injury and generally requires immediate reconstruction. Accordingly, contemporary VS surgery relies on systematic intraoperative facial nerve monitoring to support functional preservation. Current evidence-based guidelines recommend intraoperative facial nerve monitoring during VS microsurgery; commonly used strategies include free-running electromyography and direct electrical stimulation, while facial motor evoked potentials and other continuous monitoring techniques may provide complementary information. Nevertheless, no single monitoring modality reliably predicts long-term facial function, and postoperative recovery remains influenced by both tumor- and nerve-related factors [4]. Postoperative facial nerve palsy (FNP) typically manifests as unilateral weakness or paralysis of the muscles of the forehead, eyelid, and mouth, resulting in impaired speech articulation, difficulties with eating and drinking, inadequate saliva control, and ocular complications [2,5,6]. The severity of FNP ranges from mild paresis to near-complete or complete paralysis, with a highly variable functional impact. Importantly, postoperative facial dysfunction does not necessarily correlate with macroscopic preservation of facial nerve continuity. Functional impairment may arise from different degrees of neural injury, including neurapraxia, axonotmesis, or neurotmesis [7]. These injuries may result from intraoperative mechanical manipulation, traction, vascular compromise or ischemia, thermal injury, or secondary inflammatory and edematous changes affecting axonal conduction. The potential for spontaneous recovery is closely related to the type and severity of nerve damage: neurapraxia and axonotmesis frequently allow partial or complete functional recovery over time, whereas neurotmesis is usually associated with permanent deficits unless nerve repair or reconstruction is performed [7,8], with variable and often unpredictable rehabilitative outcomes. Facial nerve rehabilitation plays a central role in the postoperative management of FNP. Several interventions have been proposed to facilitate recovery, including facial motor retraining exercises, electrical stimulation, mime therapy, mirror therapy, and biofeedback techniques [9,10,11,12,13,14,15]. These approaches aim to promote neural plasticity, optimize motor relearning, prevent secondary muscle contractures, and improve functional and aesthetic outcome.

In more severe or prolonged cases, patients may develop disabling sequelae such as facial contracture, synkinesis, or hemifacial spasm, which can significantly impair social interaction, physical functioning, and emotional well-being [16]. Although patients undergoing VS surgery typically experience a relatively short hospital stay, recovery of facial nerve function is often protracted and requires structured rehabilitation and long-term follow-up. The aim of the present study is to characterize the pattern and timing of facial nerve recovery in patients developing FNP after VS surgery. A clearer understanding of the temporal course of recovery may improve prognostic counseling, guide longitudinal assessment and rehabilitation planning, and help identify patients at risk of persistent functional impairment.

## 2. Materials and Methods

### 2.1. Population

The study was conducted in accordance with the ethical principles of the Declaration of Helsinki and was approved by the Ethics Committee of University Hospital of Padova (protocol number: 476n/AO/24). Data were examined in compliance with Italian privacy and sensitive data laws. The research was a retrospective observational analysis based exclusively on fully anonymized clinical data. All data was collected during routine patient care, without any additional procedures or interventions performed for research purposes, and patient confidentiality was strictly maintained. Data were collected and reported according to the STROBE statement.

Patients undergoing surgical resection of VS between January 2020 and December 2023 at the Department of Otolaryngology–Head and Neck Surgery of the University Hospital of Padova (Italy) were retrospectively screened. The institutional surgical database, and the corresponding clinical records were reviewed to identify patients who developed postoperative facial nerve palsy. All surgical procedures were performed by the same dedicated skull base surgical team.

During the study period, 101 patients underwent VS resection, of whom 58 (57.4%) developed a variable grade of postoperative FNP.

**Figure 1:**
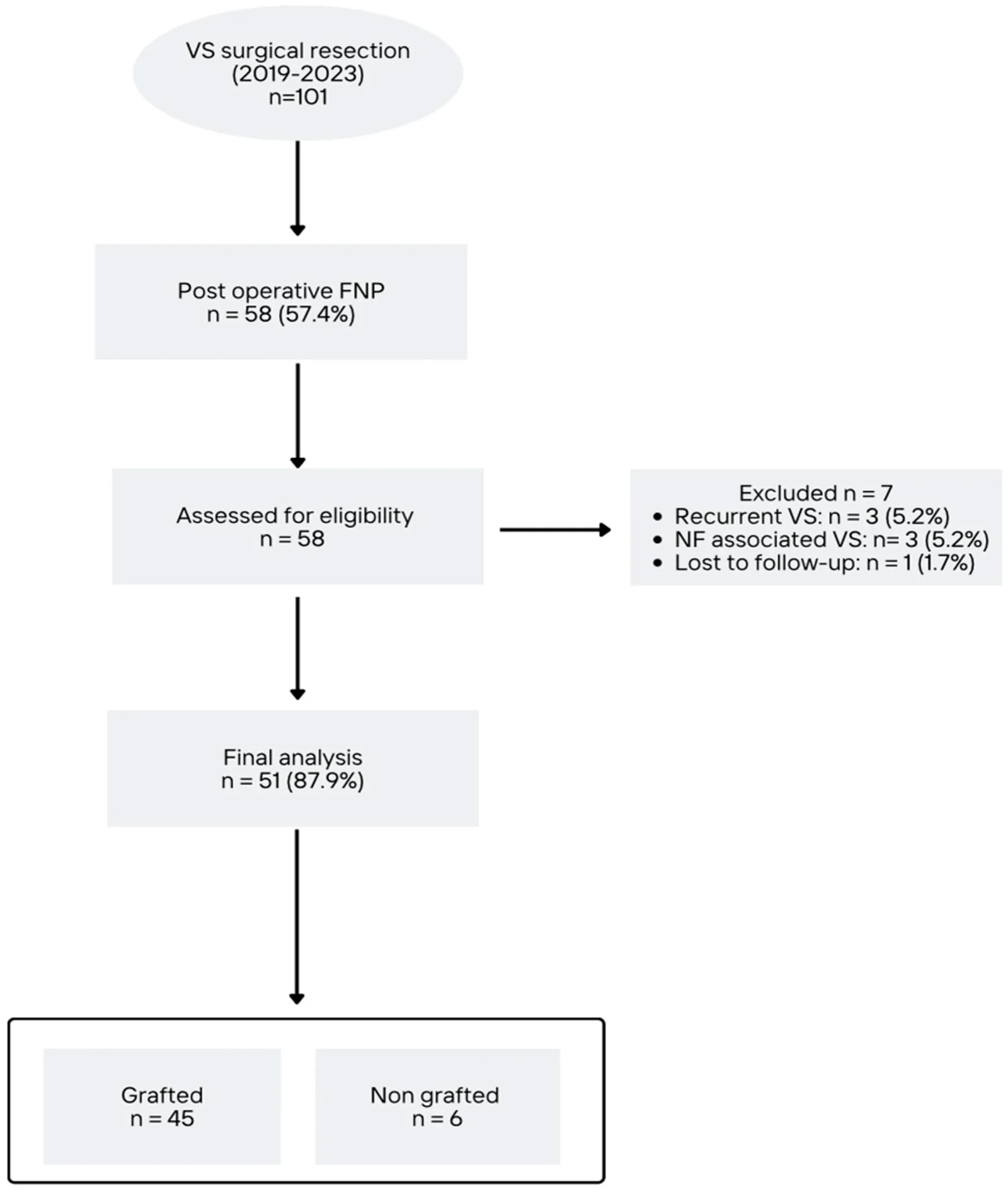
Flow diagram of cohort formation.

Inclusion criteria were: (i) age ≥ 18 years at the time of surgery; (ii) development of postoperative facial nerve palsy; (iii) at least 12 months of postoperative follow-up of 12 months conducted at the same ENT clinic. Pre-existing facial impairment was not an exclusion criterion and a worsening of the paralysis after surgery was required for patients to be included in the study. Exclusion criteria included surgery for recurrent VS and diagnosis of Neurofibromatosis type II. Of the 58 patients with postoperative FNP, three patients (5.2%) undergoing surgery for recurrent VS, three patients (5.2%) with Neurofibromatosis type II-associated VS, and one patient (1.7%) because of follow-up shorter than 12 months, resulting in a final cohort of 51 patients. A flow diagram of cohort formation is provided in Figure 1.

Surgical approach was selected according to tumor characteristics, hearing status, anatomical considerations, and surgeon preference. A translabyrinthine or retrosigmoid approach was used in the majority of cases; one patient underwent a middle cranial fossa approach and one underwent a transotic approach. The retrosigmoid approach with posterior meatotomy of the internal auditory canal was used in selected patients undergoing hearing preservation surgery [17]. Tumor size was assessed by measuring the maximum extrameatal diameter of the vestibular schwannoma, considering only the portion extending beyond the internal auditory canal and excluding the intracanalicular component.

### 2.2. Facial Nerve Assessment

At the end of VS removal, facial nerve status was classified according to the operative findings as anatomically intact, manipulated but macroscopically preserved, partially injured, or completely transected. Patients whose facial nerve was completely transected underwent intraoperative simultaneous reconstruction with autologous nerve graft. Facial nerve function was assessed using the House–Brackmann (HB) grading system [18] and the Sunnybrook Facial Grading System (SBFGS) [19,20]. Assessments were performed at hospital discharge and at 1, 3, 6, and 12 months after surgery. The HB scale ranges from grade I (normal facial function) to grade VI (complete facial paralysis). The SBFGS provides a composite score ranging from 0 to 100, based on facial symmetry at rest, symmetry during five voluntary movements, and the presence and severity of synkinesis, with higher scores indicating better facial function.

For the prevalence analysis, synkinesis was defined as an SBFGS synkinesis subscore > 0; a score of 0 was classified as absence of synkinesis. Synkinesis prevalence at each follow-up time point was therefore reported as the number and percentage of patients with a synkinesis subscore > 0.

Although the study was retrospective, HB and SBFGS assessments were obtained as part of the routine clinical follow-up pathway and retrospectively extracted for the present analysis. All 51 included patients had available HB and SBFGS assessments at discharge and at 1, 3, 6, and 12 months.

### 2.3. Facial Palsy Rehabilitation Program

Rehabilitation goals and strategies were individualized according to each patient’s functional limitations. Facial palsy was categorized into three clinical patterns: flaccid (or complete) facial palsy, active motion without synkinesis, and active motion with synkinesis [21]. During the flaccid phase, patients typically exhibit absent or minimal voluntary muscle activation on the affected side, resulting in difficulties with eye and lip closure, oral competence, mastication, saliva control, and speech articulation [22]. In this phase, rehabilitation focused primarily on soft tissue mobilization to promote circulation, reduce edema, and preserve muscle and connective tissue health.

As facial nerve regeneration progressed, patients were guided to perform slow, controlled, and symmetric facial movements, avoiding mass movements and excessive activation of the unaffected side. In patients presenting with active motion associated with synkinesis, treatment initially emphasized relaxation strategies aimed at reducing facial hypertonia, including intensive soft tissue mobilization and synkinesis control techniques. Once excessive muscle tension was reduced, movement control strategies were progressively introduced.

Regardless of facial palsy type, patient education and self-management strategies represented a core component of the rehabilitation program. Patients were educated on facial nerve anatomy and function to facilitate awareness of abnormal movement patterns and optimize adherence to rehabilitation strategies [21]. Specialized speech and language therapists evaluated patients during hospitalization, instructing them in self-administered soft tissue mobilization techniques and providing practical guidance to improve oral competence during meals. Illustrated pamphlets were provided to support home-based exercises. Patients were engaged in appointments with a speech and language therapist weekly during the first month after discharge and then monitored with monthly sessions, for a total of 15 rehabilitative sessions of 45 min each. During follow-up visits, rehabilitation strategies were progressively adapted according to functional recovery as assessed by SBFGS. The score of voluntary movement (initial movement or more) and synkinesis (mild or more) determined the introduction of voluntary movements and synkinesis management techniques. Once synkinesis was identified, the focus shifted from active movement facilitation to neuromuscular retraining, incorporating proprioceptive exercises, aimed at reducing pathological co-contractions and promoting selective motor control.

Despite the institutional facial rehabilitation pathway offered to all patients, quantitative measures of rehabilitation exposure, session frequency, home-exercise adherence, treatment intensity, and cumulative rehabilitation dose were not systematically available in the retrospective dataset. The present study was therefore not designed to estimate the independent effect of rehabilitation on facial nerve recovery.

### 2.4. Statistical Analysis

Continuous variables were summarized as mean ± standard deviation (SD) or median and interquartile range (IQR), according to their normality (Shapiro–Wilk test), and categorical variables were reported as counts and percentages.

Between-group comparisons were performed using Welch’s *t*-test or the Mann–Whitney U test for continuous variables, as appropriate, and Fisher’s exact test for categorical variables when sparse cell counts were present.

Longitudinal changes in HB grade, total SBFGS score, resting symmetry, voluntary movement, and synkinesis were evaluated across discharge and 1-, 3-, 6-, and 12-month assessments using the Friedman test. Kendall’s coefficient of concordance (W) was calculated as an overall effect-size measure. When appropriate, post hoc paired comparisons were performed using Wilcoxon signed-rank tests with Bonferroni adjustment within each outcome-specific family of comparisons, and effect sizes (r) were calculated for the principal paired comparisons. For selected clinically relevant within-patient changes, 95% confidence intervals were estimated using non-parametric bootstrap resampling with 5000 replicates. No additional multiplicity adjustment was applied across the different facial function outcomes. A favorable facial nerve outcome at 12 months was defined consistently as HB grade I–II, whereas HB grade III–VI was classified as unfavorable. Given the limited number of unfavorable outcomes (14/51), covariate selection for multivariable analysis was deliberately parsimonious and based on clinical relevance rather than data-driven stepwise selection. Age and tumor size were selected as baseline adjustment covariates, while early postoperative facial nerve function was the principal variable of interest. Because HB grade and SBFGS score at discharge assess overlapping aspects of early postoperative facial function, their relationship was evaluated using Spearman’s rank correlation coefficient. Owing to the strong inverse correlation observed between these measures, they were not entered simultaneously. Two separate Firth-penalized logistic regression models were therefore fitted: Model A included age, tumor size, and HB grade at discharge; Model B included age, tumor size, and SBFGS score at discharge. SBFGS was rescaled so that the corresponding odds ratio represents a 10-point increase. Firth penalization was used to reduce small-sample bias and provide finite estimates in the presence of separation. Grafting was not included in the primary multivariable models because only six patients underwent grafting and all six had an unfavorable 12-month outcome, resulting in complete separation. Its adjusted association was explored separately in Firth-penalized sensitivity analyses and interpreted as exploratory. Regression results are reported as odds ratios (ORs) with profile penalized-likelihood 95% confidence intervals (CIs) and *p* values. All 51 patients had complete longitudinal HB and SBFGS data and were included in the Friedman analyses without imputation. Tumor size was unavailable in 10 patients; consequently, multivariable models including tumor size were based on 41 complete cases. A two-sided *p* value < 0.05 was considered statistically significant. Statistical analyses were performed using RStudio (version 4.3.1, https://www.rstudio.com/ R Foundation for Statistical Computing, Vienna, Austria).

## 3. Results

All 51 patients had complete HB and SBFGS assessments at each prespecified longitudinal time point. Tumor size was available in 41/51 patients (80.4%). The study cohort comprised 26 males (50.9%) and 25 females (49.1%). Tumor laterality was right-sided in 20 cases (39.2%) and left-sided in 31 cases (60.8%). The mean age at surgery was 58.4 ± 11.1 years (range, 23–76 years). Among the 41 patients with available tumor-size measurements, median tumor size was 18.0 mm (IQR 10.0–27.0). According to the Koos classification, tumors were graded as Koos I in 10 patients (19.6%), Koos II in 15 patients (29.4%), Koos III in 14 patients (27.5%), and Koos IV in 12 patients (23.5%). An intracanalicular component was present in 49 cases (96.1%), while brainstem compression was observed in 11 cases (21.6%). A cystic component was identified in 16 cases (31.4%).

Surgical resection was performed via a translabyrinthine approach in 43 patients (84.2%), a retrosigmoid approach in 6 patients (11.8%), a middle cranial fossa approach in 1 patient (2.0%), and a transotic approach in 1 patient (2.0%). The mean postoperative hospital stay was 11.2 ± 4.5 days. Preoperative facial nerve function was normal (HB grade I) in 42 patients (82.4%) and mildly impaired (HB II) in 9 patients (17.6%).

At the end of surgery, the facial nerve was anatomically intact in 40 patients (78.4%), manipulated but macroscopically preserved in 4 patients (7.8%), partially injured in 3 patients (5.9%), and completely transected in 4 patients (7.8%). All four patients with complete transection underwent reconstruction with an autologous greater auricular nerve graft. Among the three patients with partial structural injury, two underwent graft reconstruction, whereas the remaining patient was judged to have sufficient residual nerve continuity and did not undergo grafting. Overall, six patients (11.8%) underwent facial nerve graft reconstruction. Five reconstructions were performed as interposition graft repairs with end-to-end graft-to-nerve anastomoses, whereas one was performed using a bypass configuration, in which an end-to-side interposed nerve grafting has been done as a reinforcement strategy to enhance axonal regeneration while preserving residual nerve continuity. The greater auricular nerve was used as the donor graft in all cases.

Additional treatments during follow-up included botulinum toxin in 6 patients (11.8%), gold-weight implantation in 4 (7.8%), lateral tarsal strip surgery in 1 (2.0%), and upper-eyelid blepharoplasty in 1 (2.0%). Demographic/tumor and surgical/postoperative characteristics are summarized separately in Table 1 and Table 2.

### 3.1. Facial Nerve Outcomes

The longitudinal evolution of facial nerve function assessed by HB grading and the SBFGS at 1, 3, 6, and 12 months after surgery is summarized in Table 3.

### 3.2. Temporal Evolution of Facial Nerve Function

**Figure 2:**
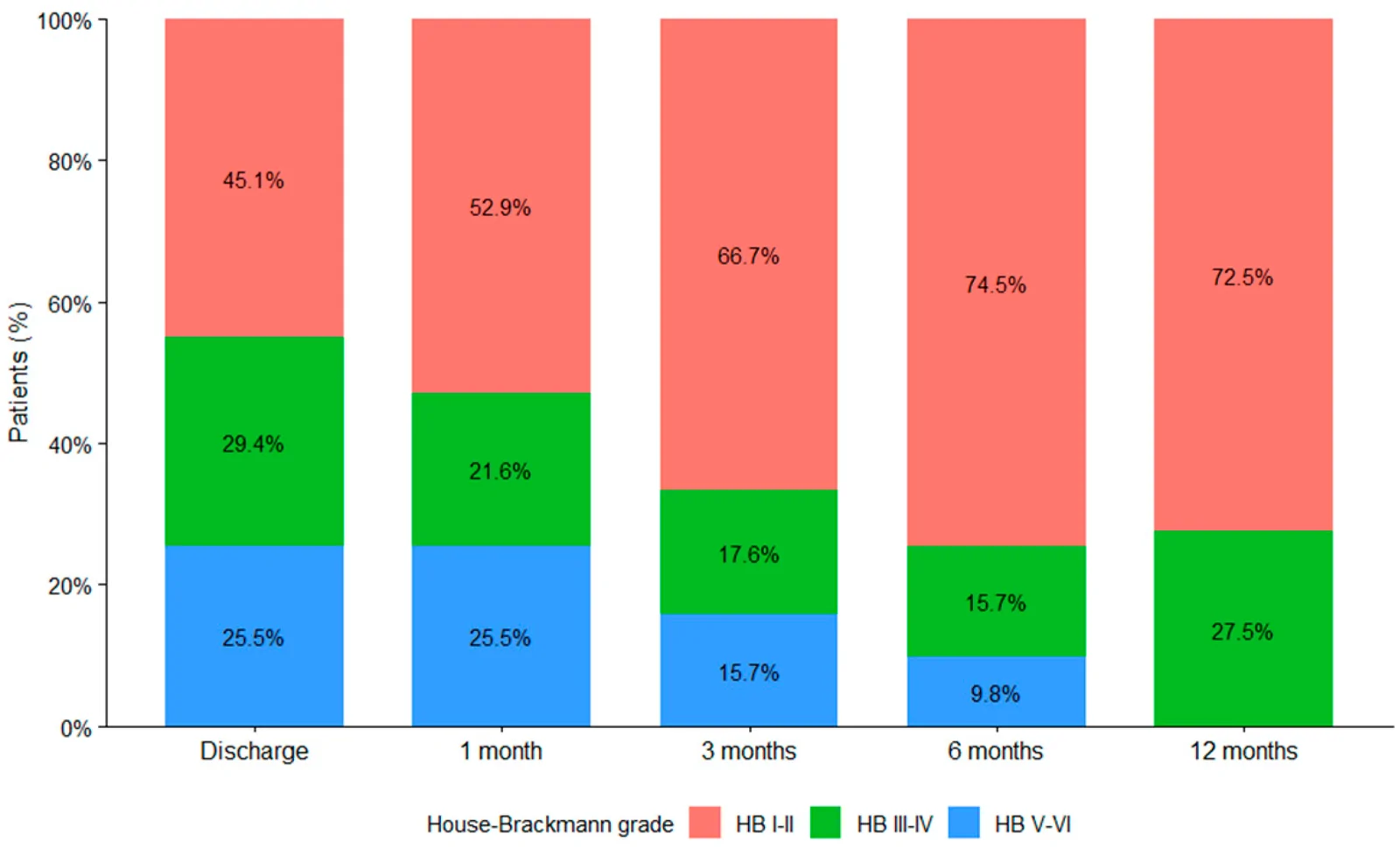
Longitudinal distribution of House–Brackmann (HB) grades over follow-up. Bars represent the proportion of patients in each HB category at discharge, 1, 3, 6, and 12 months.

**Figure 3:**
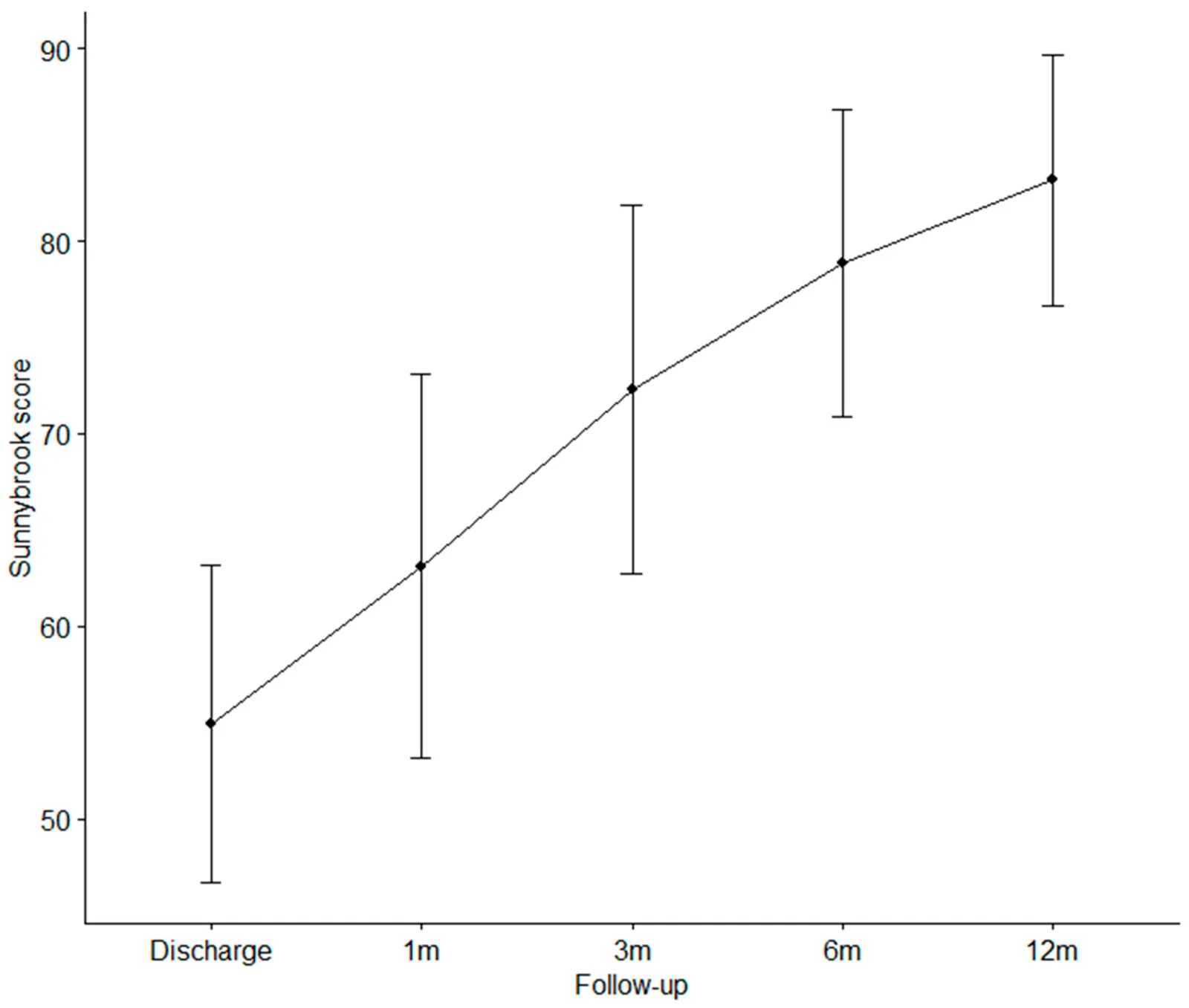
Longitudinal changes in Sunnybrook Facial Grading System (SBFGS) total scores. Points represent median values at discharge and at 1, 3, 6, and 12 months after surgery; error bars represent the interquartile range (25th–75th percentile). All time points include 51 patients.

At hospital discharge, 23 patients (45.1%) had HB grade II, 15 (29.4%) had HB III–IV, and 13 (25.5%) had HB V–VI. HB grade improved significantly throughout follow-up (Friedman χ^2^ = 107.48, df = 4, *p* < 0.001), with a large overall effect size (Kendall’s W = 0.53). At 12 months, 37/51 patients (72.5%) had achieved HB I–II, 14/51 (27.5%) had HB III–IV, and no patient remained in the HB V–VI category (Figure 2). Total SBFGS score also improved significantly over time (Friedman χ^2^ = 118.78, df = 4, *p* < 0.001; Kendall’s W = 0.58), increasing from a median of 58.0 (IQR 30.0–82.5) at discharge to 99.0 (IQR 67.5–100.0) at 12 months (Figure 3). The median within-patient improvement between discharge and 12 months was 26 points (IQR 12.5–42.0; bootstrap 95% CI 18–33). Post hoc pairwise comparisons confirmed significant improvement from discharge to 3, 6, and 12 months after Bonferroni correction (all adjusted *p* < 0.001). Component-specific SBFGS analyses also demonstrated significant longitudinal changes in resting symmetry (Friedman χ^2^ = 69.44, df = 4, *p* < 0.001; Kendall’s W = 0.34), voluntary movement (χ^2^ = 115.65, df = 4, *p* < 0.001; W = 0.57), and synkinesis (χ^2^ = 48.02, df = 4, *p* < 0.001; W = 0.24). The largest improvement was observed in voluntary movement, whereas resting symmetry improved early and remained stable during follow-up (Table 3). The median synkinesis subscore remained 0 throughout follow-up because of the highly zero-inflated distribution. However, when synkinesis was defined as an SBFGS synkinesis subscore > 0, its prevalence increased from 1/51 (2.0%) at discharge to 2/51 (3.9%) at 1 month, 1/51 (2.0%) at 3 months, 10/51 (19.6%) at 6 months, and 19/51 (37.3%) at 12 months (Table 3).

### 3.3. Subgroup Analysis: Patients Undergoing Facial Nerve Grafting

Six patients underwent immediate facial nerve graft reconstruction. Given the small subgroup size, these analyses were considered exploratory. Median HB grade changed from 5.0 (IQR 5.0–5.0) at discharge to 3.0 (IQR 3.0–3.75) at 12 months, while median SBFGS increased from 17.5 (IQR 10.0–28.0) to 46.5 (IQR 39.3–56.8). Longitudinal changes were observed for both HB grade (Friedman χ^2^ = 12.66, df = 4, *p* = 0.013) and total SBFGS score (χ^2^ = 12.61, df = 4, *p* = 0.013). These findings should be interpreted cautiously because of the scarce numerosity. Individual intraoperative characteristics, reconstruction details, longitudinal outcomes, and additional treatments are provided in Supplementary Table S1.

Longitudinal analysis demonstrated a significant change in HB grading over time (Friedman test: χ^2^ = 12.66, df = 4, *p* = 0.013). A significant improvement between discharge and 12 months was also observed using the paired Wilcoxon signed-rank test (*p* = 0.031). Similarly, SBFGS scores showed a significant increase between discharge and the 12-month follow-up (paired Wilcoxon signed-rank test, *p* = 0.031).

### 3.4. Factors Associated with Facial Nerve Recovery

A favorable 12-month facial nerve outcome, defined as HB grade I–II, was observed in 37/51 patients (72.5%), whereas 14/51 (27.5%) had HB grade III–VI. In univariable Firth-penalized logistic regression, larger tumor size was associated with lower odds of favorable 12-month facial nerve function (OR 0.92 per 1 mm increase, 95% CI 0.84–0.99; *p* = 0.017), as shown in Table 4. Worse HB grade at discharge was associated with lower odds of favorable outcome (OR 0.27 per one-grade increase, 95% CI 0.12–0.49; *p* < 0.001), whereas higher SBFGS score at discharge was associated with higher odds of favorable outcome (OR 1.95 per 10-point increase, 95% CI 1.42–3.10; *p* < 0.001).

HB grade and SBFGS score at discharge were strongly inversely correlated (Spearman ρ = −0.943, *p* < 0.001) and were therefore evaluated in separate multivariable models. In the HB-based model (*n* = 41), each one-grade increase in HB severity at discharge was independently associated with lower odds of favorable 12-month function (OR 0.30, 95% CI 0.11–0.58; *p* < 0.001), whereas age (OR 1.06, 95% CI 0.97–1.18; *p* = 0.201) and tumor size (OR 0.92, 95% CI 0.82–1.00; *p* = 0.062) were not independently associated with the outcome.

In the alternative SBFGS-based model (*n* = 41), each 10-point increase in discharge SBFGS was independently associated with higher odds of favorable 12-month function (OR 1.90, 95% CI 1.32–3.53; *p* < 0.001), whereas age (OR 1.05, 95% CI 0.96–1.16; *p* = 0.330) and tumor size (OR 0.91, 95% CI 0.81–1.01; *p* = 0.067) were not independently associated with the outcome.

All six graft recipients had an unfavorable 12-month outcome according to the prespecified HB I–II threshold, resulting in complete separation. Firth-penalized sensitivity analyses including grafting generated highly imprecise estimates (HB-based model: OR 0.10, 95% CI 0.0006–1.60, *p* = 0.112; SBFGS-based model: OR 0.08, 95% CI 0.0005–1.35, *p* = 0.085). Accordingly, these analyses do not support reliable inference regarding an independent effect of grafting.

In an exploratory analysis according to year of surgery, early postoperative facial function differed across the 2020–2023 study period for both HB grade at discharge (Kruskal–Wallis χ^2^ = 12.10, df = 3, *p* = 0.007) and SBFGS score at discharge (χ^2^ = 11.53, df = 3, *p* = 0.009). However, no significant differences by surgical year were observed for HB grade at 12 months (*p* = 0.140), SBFGS score at 12 months (*p* = 0.119), or the proportion of patients achieving HB I–II at 12 months (Fisher’s exact test, *p* = 0.433).

## 4. Discussion

Postoperative FNP remains a major concern following VS resection, despite advances in microsurgical techniques and intraoperative monitoring [1]. In this study, 51 patients who developed FNP after VS surgery were systematically evaluated over a 12-month period, providing a detailed characterization of the temporal evolution of facial nerve recovery using both the HB grading system and the SBFGS [23].

Despite substantial clinical interest in facial nerve outcomes following VS resection, the longitudinal trajectory of recovery remains poorly characterized, as most published series rely on single time-point assessments that cannot distinguish transient from permanent deficits. The present study addresses these gaps through three key methodological features: (i) sequential outcome reporting at predefined postoperative intervals, enabling a dynamic rather than static characterization of recovery; (ii) a dual grading approach integrating the House–Brackmann scale and the Sunnybrook Facial Grading System, whose complementary strengths enhance both sensitivity and cross-study comparability, integrating longitudinal analyses of both global and component-specific facial function, including resting symmetry, voluntary movement, and synkinesis; and (iii) a dedicated analysis of the facial nerve grafting subgroup, a population frequently underrepresented in the literature. Longitudinal evaluation revealed a consistent trend of functional recovery throughout the first postoperative year, with 72.5% of patients achieving HB grades I–II and a median SBFGS total score of 99 at 12 months. Although these findings indicate excellent overall functional recovery, the high median SBFGS score may also reflect a ceiling effect of the instrument, which grades overall hemifacial function on a 100-point scale [19,20]. Indeed, the temporal evolution was not uniform across facial function domains: while motor function progressively improved, synkinesis became increasingly prevalent during later follow-up. This divergence highlights why composite scores alone may incompletely characterize facial recovery and supports the combined assessment of global function, domain-specific impairment, and synkinesis.

Notably, no patient exhibited severe facial dysfunction (HB V–VI) at the end of follow-up, and synkinesis emerged predominantly after 3–6 months, reflecting the delayed maturation of aberrant reinnervation pathways. These findings are in line with previous reports demonstrating gradual but substantial recovery of facial function following VS surgery: specifically, Jia et al. [24] describe 80% complete recovery at 1 year after surgery, while Tatagiba et al. [25] reported 90% HB I–II recovery at 15 months. Aysel et al. [26] reported that greater initial severity predicted worse outcomes in Bell’s palsy, and similar results were observed in parotidectomy patients [27], suggesting that early functional severity represents a generalizable prognostic principle across etiologically distinct forms of peripheral facial palsy. However, several methodological and case-mix differences warrant careful consideration when interpreting this comparison.

Although the magnitude of SBFGS improvement was substantial, statistical significance should not be equated automatically with patient-perceived clinical benefit. A validated minimal clinically important difference for the SBFGS has not been established specifically for postoperative facial palsy after VS surgery. The improvement in composite SBFGS was nevertheless accompanied by concordant changes in HB grade, resting symmetry, and voluntary movement, suggesting substantial functional recovery. However, synkinesis developed in approximately 37% of patients, emerging predominantly between 3 and 6 months postoperatively. The underlying pathophysiology involves misrouted axonal regeneration, polyneuronal innervation, disruption of facial nucleus somatotopic organization, and cortical reorganization-mechanisms comprehensively reviewed by Rail et al. [28]. Notably, synkinesis emergence was associated with a relative reduction in SBFGS scores despite concurrent improvement in voluntary movement, reflecting the instrument’s sensitivity to aberrant reinnervation—a dimension entirely absent from HB-based assessments. As reported by Rail et al. [28], onset may occur up to 40 months after injury, underscoring the need for extended follow-up beyond the 12-month horizon of the present study. From a therapeutic standpoint, synkinesis remained manageable in the majority of cases: early structured rehabilitation likely attenuated its severity, while botulinum toxin chemodenervation was required in 6 (11.8%) patients [28]. Although patient-reported outcome measures (PROMs) are reported to be a valuable measure, they were not systematically collected in the present study and the clinical importance of the observed change cannot be formally quantified from the present dataset. The recovery occurred within a multidisciplinary postoperative pathway that included facial rehabilitation [22]. However, all patients were offered rehabilitation, no untreated comparison group was available, and quantitative measures of treatment exposure, intensity, adherence, and cumulative dose were not systematically captured. Therefore, the present study cannot isolate the independent contribution of rehabilitation or establish a causal relationship between the rehabilitation program and facial nerve recovery. Prospective studies with standardized treatment exposure and adherence measures are required to evaluate this question.

Patients undergoing facial nerve grafting represented a clinically distinct subgroup characterized by severe structural nerve injury. Descriptively, both HB and SBFGS measures improved during follow-up, consistent with the prolonged time required for axonal regeneration after nerve reconstruction [28]. However, only six patients underwent grafting and none achieved the prespecified favorable outcome of HB I–II at 12 months. Consequently, subgroup inferential analyses have very limited statistical power, and the present data cannot establish the independent effect of grafting or support comparisons with patients whose facial nerve remained anatomically continuous. Patient-level reporting is therefore more informative than aggregate comparative estimates for this subgroup. Early postoperative facial function was independently associated with 12-month outcome in both parsimonious penalized regression models. Because discharge HB and SBFGS scores were highly correlated, they were analyzed separately, and both showed consistent associations with subsequent recovery after adjustment for age and tumor size. Accordingly, facial nerve function at discharge, assessed by either HB or SBFGS, emerged as the strongest variable independently associated with favorable outcome at 12 months. This finding is consistent with the observations of Jia et al. [24], who reported that all patients with normal immediate postoperative facial nerve function recovered to HB grade I at 1-year follow-up, further supporting the prognostic value of early postoperative assessment as a reliable surrogate marker of long-term functional outcome. These findings support the prognostic relevance of early postoperative functional assessment; however, the analyses should be regarded as exploratory given the limited number of unfavorable outcomes and the complete-case sample of 41 patients used in the adjusted models. The study was not designed to compare the predictive performance of HB and SBFGS, and neither measure should therefore be interpreted as superior on the basis of these analyses.

An exploratory analysis by year of surgery identified differences in facial function at discharge during the 2020–2023 study period, whereas 12-month HB and SBFGS outcomes did not differ significantly across surgical years. The causes of these early differences cannot be determined from the retrospective dataset and may include variation in case mix, intraoperative nerve injury, surgical practice, rehabilitation, or other temporal factors. In particular, the present analyses cannot attribute these differences to pandemic-related disruptions during 2020–2021. The small number of patients within individual years further limits interpretation of temporal trends.

These findings may inform postoperative counseling by illustrating that facial function can continue to evolve throughout the first postoperative year and that early postoperative dysfunction does not necessarily represent the final functional state. Domain-specific assessment may also help clinicians identify emerging synkinesis and tailor supportive management during follow-up. The results should, however, be interpreted as descriptive and prognostic rather than evidence for the effectiveness of any specific rehabilitation strategy.

This study has several limitations. First, its retrospective, single-center design and relatively small cohort limit generalizability and may introduce selection bias. A minimum 12-month follow-up at our institution was required for inclusion; therefore, although all 51 included patients had complete longitudinal HB and SBFGS assessments, selection toward patients with complete follow-up cannot be excluded. Tumor size was unavailable in 10 patients, reducing the complete-case sample for adjusted regression analyses to 41 patients. Second, the multivariable analyses were exploratory. Only 14 unfavorable 12-month outcomes occurred, limiting the number of covariates that could be estimated reliably. Firth penalization and parsimonious model specification were used to reduce small-sample bias, but residual instability remains possible. The grafted subgroup was particularly small (*n* = 6) and demonstrated complete separation of the primary outcome; consequently, no reliable causal or independent inference regarding grafting can be made. Third, HB and SBFGS are clinician-rated scales and may be affected by interobserver variability. Because of the retrospective design, serial assessments were not necessarily performed by the same evaluator and evaluator blinding was not systematically implemented or documented. PROMS were also not systematically collected. Additionally, several patients received adjunctive interventions during the follow-up period, including botulinum toxin chemodenervation (6 patients, 11.8%) for clinically significant synkinesis and gold-weight implantation (4 patients, 7.8%) for lagophthalmos. The timing of these interventions relative to outcome assessments was variable, and their effect on subsequent SBFGS scores, particularly the synkinesis and voluntary movement subscales, cannot be fully disentangled from the natural course of recovery. SBFGS trajectories in treated patients may therefore partly reflect the pharmacological or mechanical effects of these interventions rather than spontaneous functional improvement alone. Prospective studies with systematic pre- and post-intervention assessments would be required to isolate their independent contribution to functional outcomes. Fourth, although Bonferroni adjustment was applied to post hoc comparisons within each outcome-specific family, no additional multiplicity correction was applied across the different longitudinal outcomes. An inflated overall type I error rate due to multiple testing therefore cannot be excluded, and secondary findings should be interpreted cautiously. Finally, all patients underwent a postoperative care pathway that included facial rehabilitation, but treatment intensity, adherence, timing, and cumulative rehabilitation dose were not systematically quantified and no untreated control group was available. The independent effect of rehabilitation therefore cannot be determined. Automated computer vision-based methods such as Emotrics and Auto-eFACE may improve quantitative assessment of symmetry and movement, while surface electromyography, previously investigated by our group for facial nerve functional evaluation following VS surgery [1], may provide complementary neurophysiological information.

Future prospective multicenter studies should expand sample sizes, incorporate standardized longitudinal assessment, PROMs, and more objective measures of facial function to better capture the long-term functional and psychosocial consequences of FNP.

## 5. Conclusions

This study provides a comprehensive characterization of facial nerve recovery following VS surgery in a single-center cohort, highlighting a progressive improvement in both global and component-specific facial function over 12 months. Early postoperative HB grade and SBFGS score were independently associated with 12-month facial function when evaluated in separate parsimonious penalized regression models. Synkinesis became increasingly prevalent during later follow-up despite a median subscore of zero, emphasizing the value of reporting both severity scores and prevalence. Findings regarding patients undergoing facial nerve grafting remain exploratory because of the very small subgroup. These findings provide descriptive and prognostic information on the course of postoperative facial nerve recovery and may help inform the clinical monitoring and individualized rehabilitation planning of patients with postoperative FNP, without establishing the effectiveness of any specific rehabilitation strategy. However, given the single-center design and relatively small cohort, larger, multicenter, prospective studies are needed to determine the independent effects of nerve reconstruction and to evaluate the effectiveness and potential added benefit of specific rehabilitation strategies.

## Figures and Tables

**Table 1:** Demographic and tumor characteristics.

		Non Grafted FN *n* = 45	Grafted FN *n* = 6	Total *n* = 51	*p* Value
**Age (mean)**		59.1 ± 11.4	53.2 ± 5.8	58.4 ± 11.1	0.064
**Gender**	**Male (%)**	22 (48.9%)	4 (66.7%)	26 (51.0%)	0.668
	**Female (%)**	23 (51.1%)	2 (33.3%)	25 (49.0%)	
**Side**	**Right**	20 (44.4%)	0 (0.0%)	20 (39.2%)	0.0697
	**Left**	25 (55.6%)	6 (100.0%)	31 (60.8%)	
**Size (median, IQR)**		17.0 (10.0–25.0)	24.0 (12.5–29.5)	18.0 (10.0–27.0)	0.415
**Intracanalicular component**		43 (95.6%)	6 (100.0%)	49 (96.1%)	1.000
**Brainstem compression**		8 (17.8%)	3 (50.0%)	11 (21.6%)	0.105
**Cystic component**		13 (28.9%)	3 (50.0%)	16 (31.4%)	0.362
**Preoperative FN assessment**	**HB (median, IQR)**	1.0 (1.0–1.0)	1.0 (1.0–1.8)	1.0 (1.0–1.0)	0.298
	**HB I**	38 (84.4%)	4 (66.7%)	42 (82.4%)	0.283
	**HB II**	7 (15.6%)	2 (33.3%)	9 (17.6%)	

Abbreviations: FN: facial nerve; HB: House–Brackmann.

**Table 2:** Surgical and post operative characteristics.

		Non Grafted FN *n* = 45	Grafted FN *n* = 6	Total *n* = 51	*p* Value
**Surgical approach**	**Translabyrinthine**	38 (84.4%)	5 (83.3%)	43 (84.3%)	1.000
	**Retrosigmoid**	5 (11.1%)	1 (16.7%)	6 (11.8%)	
	**Middle cranial fossa**	1 (2.2%)	0 (0.0%)	1 (2.0%)	
	**Transotic**	1 (2.2%)	0 (0.0%)	1 (2.0%)	
**Intraoperative FN status**	**Intact**	40 (88.9%)	0 (0.0%)	40 (78.4%)	<0.001
	**Manipulated**	4 (8.9%)	0 (0.0%)	4 (7.8%)	
	**Partially damaged**	1 (2.2%)	2 (33.3%)	3 (5.9%)	
	**Transected**	0 (0.0%)	4 (66.7%)	4 (7.8%)	
**Adjunctive treatments**	**Botulinum**	5 (11.1%)	1 (16.7%)	6 (11.8%)	0.580
	**Gold weight**	0 (0.0%)	4 (66.7%)	4 (7.8%)	<0.001
	**Blepharoplasty**	1 (2.2%)	0 (0.0%)	1 (2.0%)	
	**Lateral tarsal strip**	1 (2.2%)	0 (0.0%)	1 (2.0%)	
**Hospital stay (median, IQR)**		10.0 (9.0–11.0)	10.0 (9.3–10.8)	10.0 (9.0–11.0)	0.988

Abbreviations: FN: facial nerve.

**Table 3:** Longitudinal evolution of facial nerve function assessed by HB grading and the SBFGS at 1, 3, 6, and 12 months after surgery.

		FN Evaluation					*p*-Value
		At Discharge Median (IQR)	1-Month Median (IQR)	3-Month Median (IQR)	6-Month Median (IQR)	12-Month Median (IQR)	
**HB grading system**		3.0 (2.0–4.5)	2.0 (1.5–4.5)	2.0 (1.0–3.5)	2.0 (1.0–2.5)	1.0 (1.0–3.0)	<0.001
**SBFGS**	**Total SBFGS**	58.0 (30.0–82.5)	75.0 (24.0–98.0)	90.0 (39.0–100.0)	95.0 (58.0–100.0)	99.0 (67.5–100.0)	<0.001
	**Rest SBFGS**	10.0 (5.0–10.0)	5.0 (0.0–10.0)	5.0 (0.0–10.0)	0.0 (0.0–10.0)	0.0 (0.0–5.0)	<0.001
	**Movement SBFGS**	68.0 (40.0–88.0)	80.0 (32.0–100.0)	92.0 (44.0–100.0)	96.0 (68.0–100.0)	100.0 (76.0–100.0)	<0.001
	**Synkinesis**	0.0 (0.0–0.0)	0.0 (0.0–0.0)	0.0 (0.0–0.0)	0.0 (0.0–0.0)	0.0 (0.0–2.0)	<0.001
**Patients with synkinesis**		1 (2.0%)	2 (3.9%)	1 (2.0%)	10 (19.6%)	19 (37.3%)	<0.001

Abbreviations: FN: facial nerve; HB: House–Brackmann; SBFGS: SunnyBrook Facial Grading System. Note: Synkinesis was defined as an SBFGS synkinesis subscore > 0.

**Table 4:** Firth-penalized logistic regression models for favorable facial nerve outcome (HB I–II) at 12 months.

Model 1—HB	OR	95% CI	*p*
Age, per year	1.06	0.97–1.18	0.201
Tumor size, per mm	0.92	0.82–1.00	0.062
HB at discharge, per grade	0.30	0.11–0.58	<0.001
**Model 2—SBFGS**			
Age, per year	1.05	0.96–1.16	0.330
Tumor size, per mm	0.91	0.81–1.01	0.067
SBFGS at discharge, per 10 points	1.91	1.32–3.52	<0.001

OR are derived from Firth-penalized logistic regression with profile penalized-likelihood 95% CIs. Model 1 includes age, tumor size, and HB grade at discharge. Model 2 includes age, tumor size, and SBFGS score at discharge. The SBFGS OR is expressed per 10-point increase. Both models were based on 41 complete cases.

## Data Availability

The data presented in this study are available on request from the corresponding author.

## Supplementary Materials

The following supporting information can be downloaded at https://www.mdpi.com/article/10.3390/medicina62081614/s1, Table S1: Individual characteristics and outcomes of patients undergoing facial nerve grafting.

## Abbreviations

The following abbreviations are used in this manuscript:

CI	Confidence Interval
FNP	Facial Nerve Palsy
HB	House–Brackmann
IQR	Interquartile Range
OR	Odds Ratio
SBFGS	SunnyBrook Facial Grading System
SD	Standard Deviation
VS	Vestibular schwannoma
