# Supplementary material for: Facial Nerve Palsy Recovery After Vestibular Schwannoma Surgery: Temporal Patterns and Early Predictors of Functional Outcome
Source: Medicina (Kaunas). 2026 Aug 21;62(8):1614. doi: 10.3390/medicina62081614 (PMC13515253; doi:10.3390/medicina62081614)
Supplement: Supplementary file 1 [file medicina-62-01614-s001.zip › medicina-4439118-supplementary.pdf]

Supplementary Table S1. Individual characteristics and outcomes of patients undergoing facial nerve grafting

Case	Intraoperative FN status	Reconstruction	Graft	Graft length	Discharge HB	Discharge SBFGS	12-mo HB	12-mo SBFGS	Additional treatment
1	Transected	End-to-end	GAN	NA	5	9	4	33	treatment
2	Transected	End-to-end	GAN	NA	4	38	3	50	Gold weight
3	Partial injury	End-to-end	GAN	NA	6	5	4	38	treatment
4	Transected	End-to-end	GAN	45	5	30	3	68	Gold weight
5	Transected	End-to-end	GAN	NA	5	13	3	59	Gold weight
6	Partial injury	Bypass	GAN	NA	5	22	3	43	none
